# Tissue engineering and regenerative medicine strategies for the female breast

**DOI:** 10.1002/term.2999

**Published:** 2019-12-30

**Authors:** Claudio Conci, Lorenzo Bennati, Chiara Bregoli, Federica Buccino, Francesca Danielli, Michela Gallan, Ereza Gjini, Manuela T. Raimondi

**Affiliations:** ^1^ Department of Chemistry, Materials and Chemical Engineering “Giulio Natta” Politecnico di Milano Milan Italy

**Keywords:** breast reconstruction, cell‐assisted lipotransfer, conventional therapy, regenerative therapy

## Abstract

The complexity of mammary tissue and the variety of cells involved make tissue regeneration an ambitious goal. This review, supported by both detailed macro and micro anatomy, illustrates the potential of regenerative medicine in terms of mammary gland reconstruction to restore breast physiology and morphology, damaged by mastectomy. Despite the widespread use of conventional therapies, many critical issues have been solved using the potential of stem cells resident in adipose tissue, leading to commercial products. in vitro research has reported that adipose stem cells are the principal cellular source for reconstructing adipose tissue, ductal epithelium, and nipple structures. In addition to simple cell injection, construct made by cells seeded on a suitable biodegradable scaffold is a viable alternative from a long‐term perspective. Preclinical studies on mice and clinical studies, most of which have reached Phase II, are essential in the commercialization of cellular therapy products. Recent studies have revealed that the enrichment of fat grafting with stromal vascular fraction cells is a viable alternative to breast reconstruction. Although in the future, organ‐on‐a‐chip can be envisioned, for the moment researchers are still focusing on therapies that are a long way from regenerating the whole organ, but which nevertheless prevent complications, such as relapse and loss in terms of morphology.

## 1 INTRODUCTION

### 1.1 Macroscopic anatomy

**Figure 1:**
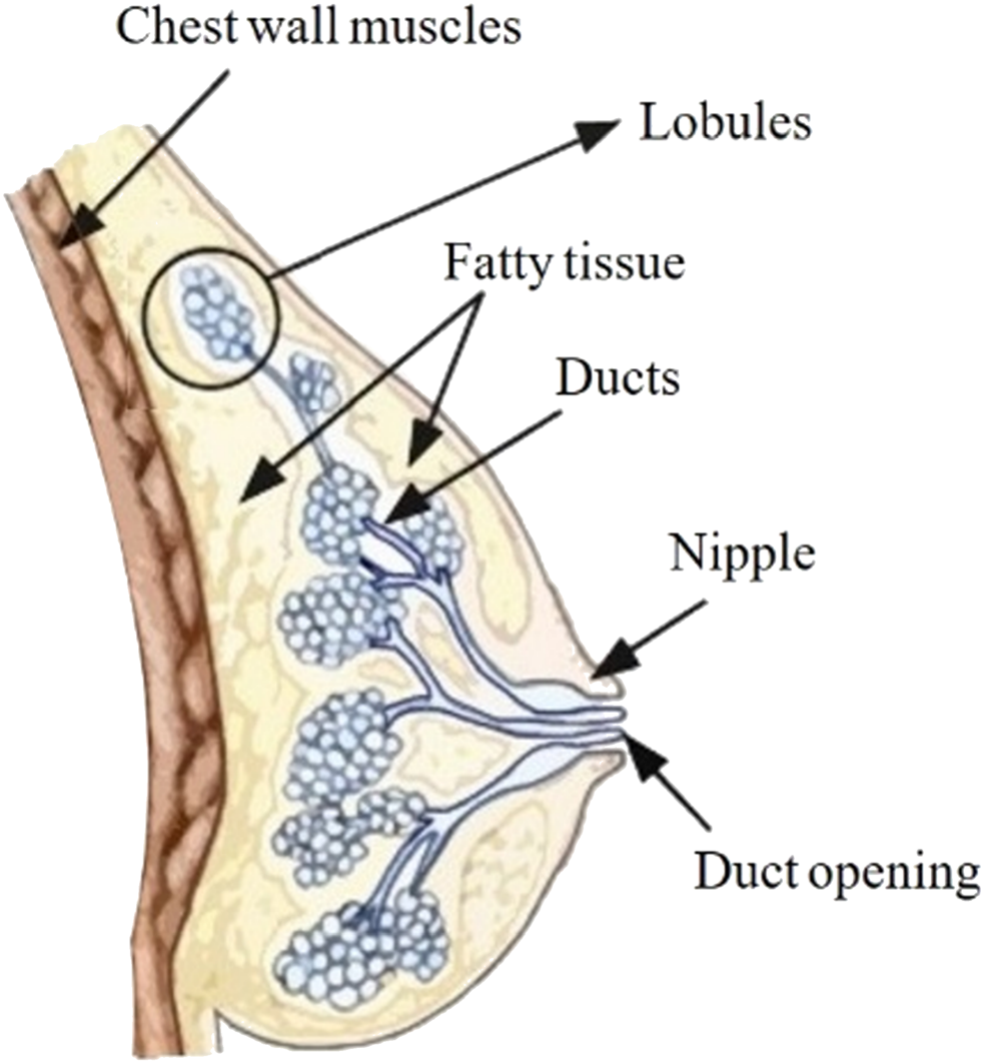
Macroscopic anatomy of an adult female breast; fat tissue characterizes the majority of the female breast and it embodies ducts and lobules ending in the nipple

Together with the adipose tissue, the mammary gland contributes the connective tissue and other minor components, to form the breast. The mammary gland distinguishes mammals from other animals and appears as tissue in the shape of a conical disk. It is present in both females and males, but only reaches complete development in females (Hassiotou & Geddes, 2013). The breast lies on the deep pectoral fascia and varies in shape and size. In an adult female, its base extends vertically from the second or third rib to the sixth rib and, into the transverse plane, from the sternal edge medially almost to the midaxillary line laterally. The mammary gland consists of between 15 and 20 lobes immersed in adipose tissue and buried by connective laminae, organized into a three‐dimensional network (Figure 1). Each lobe, which contains alveoli arranged in bunches, ends in a lactiferous duct that generally has an independent outlet on the nipple (Figure 1).

Each duct has a dilated portion, the lactiferous sinuses, in which milk accumulates during lactation. The areola (the disk of skin that circles the base of the nipple) contains sebaceous glands, which increase in size during pregnancy. The nipples are almost conical prominences containing smooth muscles without adipose tissue positioned in the center of the areolas. The overall position of the nipples is related to the stage of pregnancy. A vascularized compact fibro‐adipose tissue surrounds the glands and lactiferous ducts (Gilroy, Ross, MacPherson, & Gaudio, 2014).

### 1.2 Physiology

The development of the mammary gland is based on several stages. During the lifetime of a female, the adult mammary gland is subjected to a cyclic expansion due to hormonal changes within the menstrual cycle, lactation after pregnancy and involution (Hassiotou & Geddes, 2013). The specific function of the gland is to synthetize, secrete and deliver milk to the infant for his/her nourishment, protection, and development (Hassiotou & Geddes, 2013). The oxytocin produced in response to the suckling infant causes the surrounding myoepithelial cells to contract in order to move the milk through the ductal tree to reach the nipple (Ercan, van Diest, & Vooijs, 2011).

### 1.3 Microscopic anatomy

Multiple cell types make up the mammary tissue: epithelial cells, adipocytes, fibroblasts, vascular cells, immune cells, nerves on the gland surface, and bipotent mammary stem cells (MaSCs).

**Figure 2:**
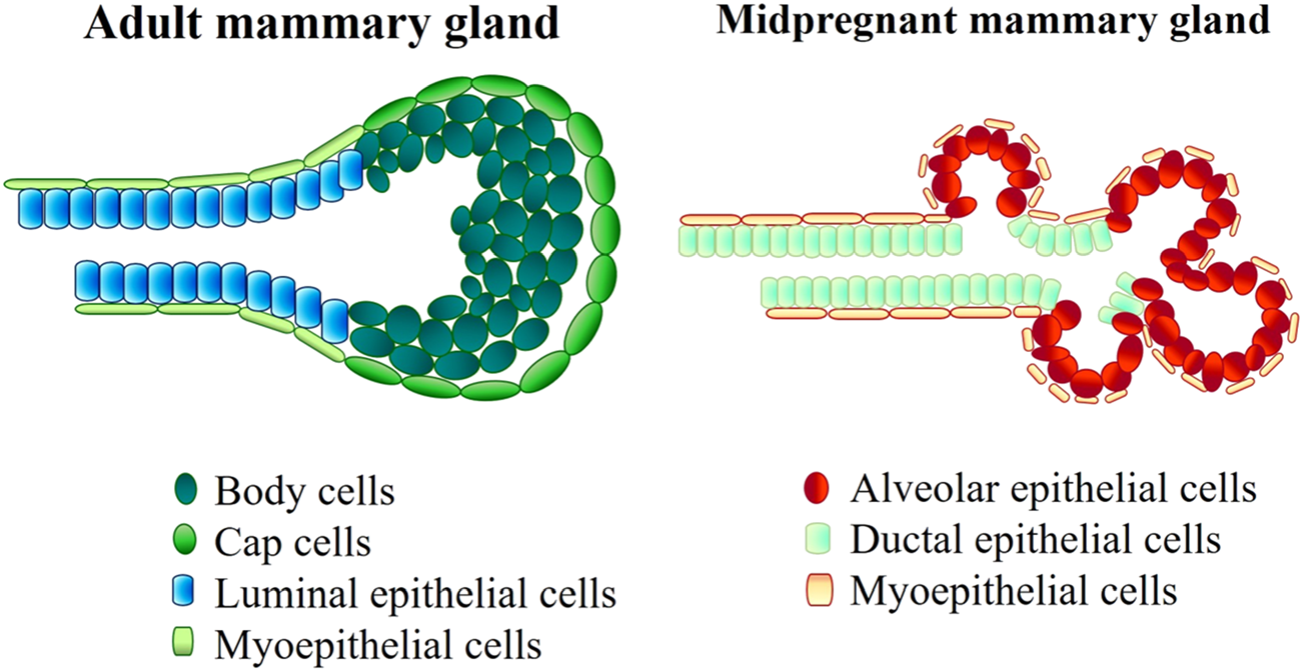
Terminal end bud. On the left, adult mammary duct is characterized by a layer of cap cells (green), which surrounds body cells (dark green). Differentiated myoepithelial and luminal epithelial cells cover the inside of the duct. On the right, midpregnant mammary gland: Myoepithelial cells are on the external side of the ducts and surround the alveolar epithelial cells (red)

The mammary epithelium shows a regenerative potential supporting the cycle of growth and involution. The epithelial compartment contains the mammary stem cells (Hassiotou & Geddes, 2013). During puberty, cap cells (multipotent stem cells; Figure 2) cover the external layer of the bud developing into myoepithelial cells or luminal epithelials, whereas body cells fill the interior part (Woodward, 2005).

Adipocytes represent the reservoir of fat needed for the metabolic process of milk production, and they influence epithelial growth and mammary epithelium function. In addition, they produce vascular endothelial growth factors and regulate mammary gland angiogenesis, thanks to the stromal vascular fraction cells (SVF). The adipose tissue surrounds the stroma, which is characterized by dense fibrous connective tissue and contains mesenchymal cells.

Fibroblasts communicate with the epithelium and produce extracellular matrix components as collagens, proteoglycans, and fibronectin in addition to enzymes. Hassiotou et al. proved the possibility of detecting, MaSCs, progenitor cells, mature myoepithelial, and milk‐secretory cells in human breast milk stem cells (Hassiotou & Geddes, 2013).

The mammary stem cells physiologically experience asymmetric cell division (Ballard et al., 2015), each subdividing into two daughters; one of which is identical to the mother and the other starts committing. This process differs from symmetric cell division, during which the mother cell two daughters identical to itself.

### 1.4 Homeostasis

In the mammary gland, homeostasis involves the renewal of somatic stem cells (basal and luminal). Ballard et al. proved that the binding between the extracellular signal SLIT2 and the cellular receptor ROBO1 controls the balance between self‐renewal based on asymmetric cell division and self‐renewal based on symmetric cell division (Ballard et al., 2015). If SLIT2/ROBO1 signaling fails, these cells tend to undergo symmetric cell division alone.

In the homeostatic process, hormones also play an important role. For example, the growth hormone, which stimulates the production of insulin‐like growth factor‐1 and estrogen (Macias & Hinck, 2012), controls homeostasis during puberty. Ovulation and the menstrual cycle then activate the MaSCs thus driving breast development. The mammary gland is completely remodeled during the pregnancy–actation cycle, due to the activation of MaSCs by a circulating hormonal complex, which leads to changes in the ductal system (Hassiotou & Geddes, 2013). In the final phase, progesterone and prolactin, two hormones responsible for the differentiation of alveoli and for the increase in secondary and tertiary ductal branching, manage gland maturation and alveologenesis (Macias & Hinck, 2012).

### 1.5 Spontaneous repair

In the case of damage or malfunction of the mammary's ductal tree, inflammation and scar formation occur. No studies have yet demonstrated the restoration of functionality, especially in the case of severe or extended injuries.

### 1.6 Pathologies requiring regenerative therapies

Most breast cancers originate in the terminal duct‐lobular unit of the mammary glands, driven by the activation of oncogenes (HER2) and the inhibition of tumor‐suppressor genes (such as BRCA1, BRCA2, and p53; Antoniou et al., 2003).

Biochemical (Gudjonsson et al., 2002) and mechanical factors can also cause tumorigenesis. The atypical presence of stomelysin‐3, around the tumor (Andarawewa et al., 2005), and the loss of the ability to produce functional laminin 332 (Kwon, Chae, Wilczynski, Arain, & Carpenter, 2012), a protein that influences cell differentiation, indicate a tumor formation through biochemical factors. On the other hand, mechanical changes in the tension of the mammary stroma indicate tumorigenesis due to the presence of mechanical factors (increased stromal collagen; Provenzano et al., 2008).

Breast cancer tumors are categorized into noncancerous (fibroadenomas, papilloma), cancerous or malignant (the fifth most common cause of death due to cancer in women, as reported by the American Institute for Cancer Research in 2018), and metastatic cancers, which involve malignant tumor cells spread locally, regionally, or nearby lymph nodes, tissues, organs, or to the distal districts of the body.

### 1.7 Conventional therapies

Several conventional therapies are currently used both for breast reconstruction, in the case of tumors, and for breast augmentation.

The most common treatment for both purposes is mastectomy—total and conservative. The latter consist of skin‐sparing mastectomy and nipple‐sparing mastectomy: Both of which are used for tumors and cosmetic purposes. A total mastectomy removes the entire breast glandular tissue. The conservative mastectomy spares the healthy skin envelope with or without the nipple‐area complex. A research group based in Ireland has shown that the remaining skin works as a pocket to facilitate the reconstruction through prosthesis (O'Halloran, Courtney, Kerin, & Lowery, 2017). Benson et al. suggested applying conservative mastectomy to women with a ductal carcinoma in situ, located at least 2 cm aside the nipple‐areola complex (NAC). On the other hand, where tumors are bigger than 5 cm, patients need to undergo a total mastectomy (Benson, Dumitru, & Malata, 2016).

A conservative mastectomy is used only in the case of cancer development and is known as breast‐conserving therapy. This treatment is a valid alternative to mastectomy, for patients with breast cancer Stages I or II, whose dimension needs to be smaller than 5 cm. The first step in this procedure, consists in breast conserving surgery, also known as a lumpectomy, aimed at eradicating the breast cancer. The second step is based on a controlled dose of radiotherapy to remove possible microscopic residuals of the tumor. Unlike mastectomy, breast conserving therapy provides both better esthetic results and reduced postoperative complications (Rahman, 2011).

The final procedure is autologous fat transfer, also called fat grafting. This is currently available for both breast reconstruction and cosmetic purposes. The core of the treatment is to harvest autologous adipose tissue, usually from the patient's abdomen or thigh and its consequent centrifugation to eliminate substances, such as lipase, which prevent the tissue from being viable for use. The processed tissue is then grafted onto the site of interest. Fat grafting may be a better alternative to the other therapies mentioned above, because adipose tissue is biocompatible, easy to obtain, and the procedure is repeatable and less expensive (Bellini, Grieco, & Raposio, 2017).

### 1.8 Critical issues regarding conventional therapies and an introduction to regenerative therapies

Synthetic prosthesis has some shortcomings, such as infection of the surrounding tissues, wound dehiscence, capsular contracture, variations in shape, dislocation from the implant site, calcification, or even a further rupture of the prosthesis itself (Galimberti et al., 2017). In the worst case, the patient requires a replacement of the implant through additional surgery. A research group based in the United States showed that almost 60% of patients who underwent breast conserving therapy, due to breast cancer, needed a re‐excision to remove remaining diseased tissue (Waljee, Hu, Newman, & Alderman, 2008). Moreover, Fajdic and colleagues, reported cases of the local recurrence of tumor after treatment (Fajdic, Djurovic, Gotovac, & Hrgovic, 2013).

Finally, regarding fat transfer, Gentile et al. reported that the percentage of surviving volume of transplanted tissue with respect to the injected tissue reached 39%. This means that the adipose tissue alone cannot naturally maintain the breast volume (Gentile et al., 2012).

As an alternative to these conventional therapies, O'Halloran and collaborators showed the increasing interest in using adipose‐derived stem cells (ADSCs) for breast reconstruction and augmentation (O'Halloran et al., 2017). These cells show a high regenerative potential and commitment mainly in mature adipocytes, which are able to maintain the viability of the transplanted adipose tissue and provide a more natural volume of the mammary gland.

## 2 IN VITRO RESEARCH

The need for greater psychological and esthetic benefits has led to the development of innovative strategies for breast reconstruction after invasive interventions, such as the mastectomy. Each newly engineered medical strategy should be modeled in order to predict the efficacy of the culture approach (Galbusera, Cioffi, & Raimondi, 2008) and then tested in vitro under different controlled conditions. Below, we summarize the various strategies that have been used in the development of the first experimental step of in vitro modeling.

### 2.1 Cell models

Embryonic stem cells are theoretically beneficial for regenerative therapy as a result of their pluripotential capacity; however because of ethical and regulatory issues, they have not been used to date. Research has thus focused on the use of primary adult ADSCs, especially autologous ones. These are multipotent cells that have been used since 2001 as a major source of cells with a regenerative potential, becoming the gold standard for regenerative techniques and breast tissue engineering (O'Halloran et al., 2017; Sterodimas, de Faria, Nicaretta, & Pitanguy, 2010).

ADSCs secrete cytokines and growth factors that stimulate tissue recovery in a paracrine manner. ADSCs modulate the host local reaction by enhancing differentiation in the endogenous stem cells along the required lineage pathway. At the same time, they provide antioxidants, free radical scavengers, and chaperone/heat shock proteins. As a result, toxic substances released into the injured environment can be removed thus promoting the recovery of the surviving cells (Gimble, Katz, & Bunnell, 2007).

There are numerous advantages of using autologous adipocytes: biocompatibility, versatility, nonimmunogenicity, and minimal donor site morbidity (Campbell, Hume, & Watson, 2014; O'Halloran et al., 2017).

ADSCs are collected by the centrifugation of the SVF, the product of liposuction, previously digested by collagenase. The SVF contains various cell types such as endothelial precursor cells, endothelial cells, pluripotent vascular progenitors, macrophages, and preadipocytes (O'Halloran et al., 2017).

The advantages of SVF composition and the easy harvest method have led to the development of strategies involving the use of ADSCs enriched with SVF (Bielli et al., 2014; Bora & Majumdar, 2017; Sterodimas et al., 2010).

In addition, the adipose tissue is heavily vascularized, providing vital networks for the normal renewal of the tissue, which thus needs to be taken into account during tissue regeneration.

Several studies have demonstrated that the interaction between vascular endothelial growth factor, secreted by ADSCs, and the endothelial precursor cells, contained in SVF, leads to a more widespread angiogenesis compared with the use of ADSCs alone, differentiated in endothelial cells (Liu, Zhuge, & Velazquez, 2009; Suga et al., 2009).

The main disadvantages of using ADSCs enriched with SVF is related to SVF's heterogeneous composition, which could lead to immunological rejection (O'Halloran et al., 2017). Numerous interactions between adipocytes and epithelial cells are still being studied, encouraging researchers to use adipocyte–epithelium cocultures (Wang, Reagan, & Kaplan, 2010).

Several studies in tissue engineering technology have shown that three‐dimensional adipocyte–epithelium cocultures enhance epithelial cell differentiation, with respect to two‐dimensional cocultures. Cell injection in the implant site is a viable alternative in clinical use; however, it has some limitations such as the formation of cysts. The positioning of a construct (cells + scaffold) has thus been studied, which could improve the benefits of breast regeneration, while limiting the drawbacks of cell administration alone. Both human adipose stem cells (hASCs) and epithelial cells involved in these studies are from human sources and thus can be labeled as primary cells.

Although the current in vitro research in adipose tissue engineering has advanced, ADSCs or adipose stem cells (ASCs) mechanisms of interaction with other physiological cell sources are still not clearly understood. ADSCs help to repair damaged or failing organs. Such mechanisms have been studied as in case of cardiac diseases, endothelial formation mechanisms (with an effective lineage commitment; Gimble et al., 2007), bone tissue, and cartilage failures (Strem et al., 2005); however, no studies to date have described how ADSCs help in the recovery of the mammary gland tissue.

Researchers have also focused on using induced pluripotent stem cells (iPSCs). This kind of cell source is obtained by converting adult somatic cells, of autologous origin, to a pluripotent phenotype by genetic engineering, such as the retroviral transduction of four specific transcription factors (Oct3/4, Sox2, Klf4, and c‐Myc; Takahashi et al., 2007).

iPSCs are usually obtained from adult fibroblasts or epithelial cells thus dramatically reducing problems related to the availability of the donor cellular source. This ability to reprogram somatic cells toward a pluripotent lineage has dramatically changed in vitro approaches in the regenerative field. Qu et al. (2017) were the first to develop a method to direct “human” iPSCs toward mammary differentiation. They cultured the cells with the MammoCult medium (Hassiotou et al., 2012), usually employed to enrich breast stem cells with nonneural ectoderm ones susceptible for the formation of mammary‐like cells, then directing the human iPSC differentiation toward the nonneural ectodermal type as a precursor of the desired tissue formation.

### 2.2 Effect of scaffold/matrix on 3‐d models

**Figure 3:**
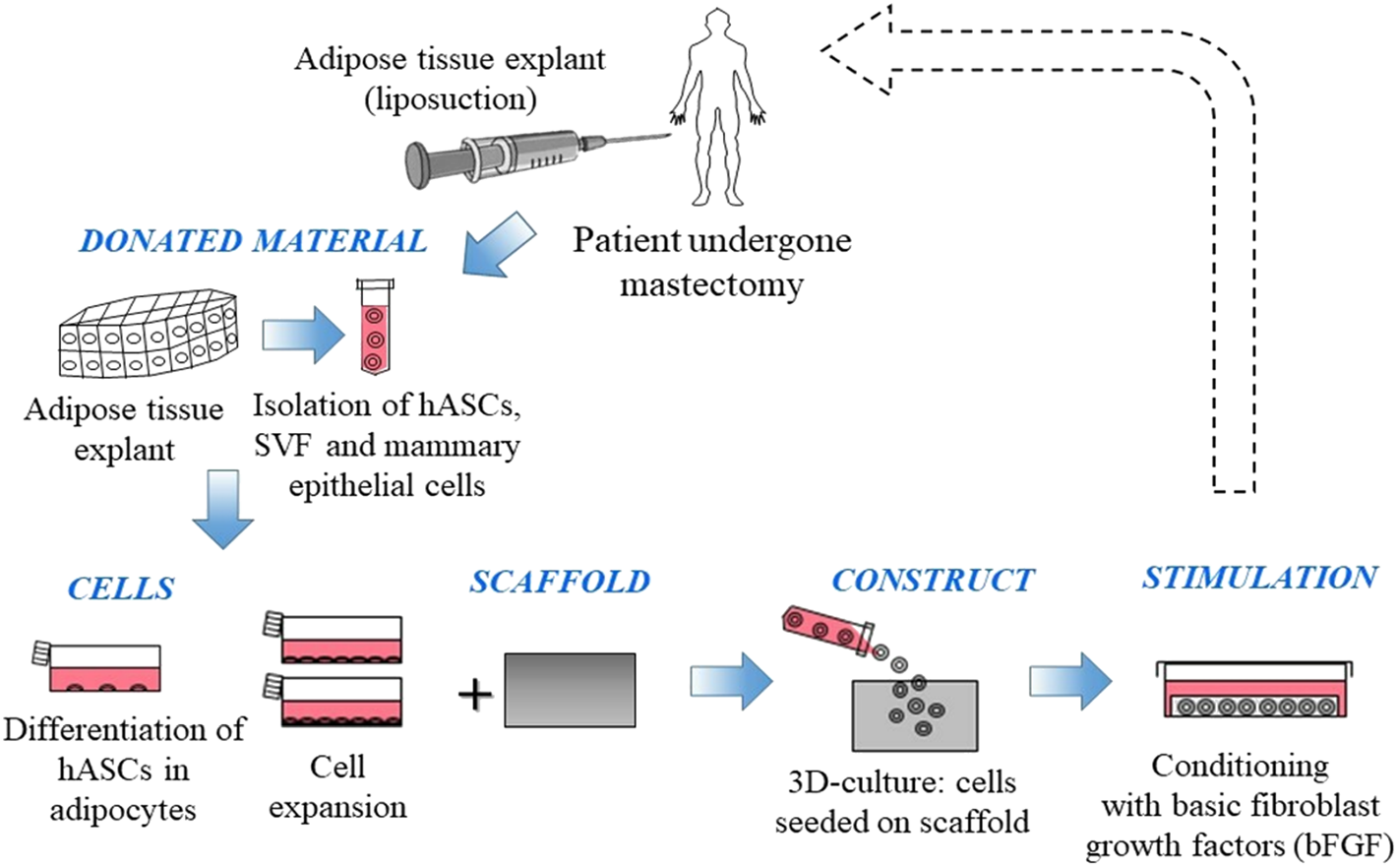
Bioengineered graft: isolation and differentiation of adipocytes from adipose tissue, cells seeding on a scaffold and suitable stimulations of the construct. The dotted line traces a possible orthotopic implantation, which does not strictly concern in vitro models, but regards clinical trials. bFGF, basic fibroblast growth factor; hASCs, human adipose stem cells; SVF, stromal vascular fraction

This section focuses on the adipose tissue regeneration. Techniques consisting in cell injection in injured sites induce retention of the tissue; thus, researchers have developed in vitro methods on the basis of cells seeded on a scaffold (O'Halloran et al., 2017; Figure 3).

Mechanical, molecular, structural cues, and scaffold properties are key (Nava et al., 2015) to maintaining the architecture and the functionality of this tissue (Nava et al., 2016; Wang et al., 2010) thus leading to ADSC proliferation and differentiation (Sterodimas et al., 2010).

For example, Tremolada and colleagues (Tremolada, Palmieri, and Ricordi, 2010) verified the expansion of hASCs in vitro: 1 g of adipose tissue provides approximately 5,000 stem cells, which is 500‐fold greater than the yield from the same volume of bone marrow. This shows that the adipogenesis capability of ASCs is greater than the other differentiation lineages when supported by a suitable 3‐D scaffold.

The choice of scaffold is thus fundamental for the success of the related regenerative therapies and for the analysis of the cell types. Examination of the types of scaffold is essential in order to get the best results from in vitro analyses.

Good scaffolds, engineered for constructing synthetic adipose tissue, require biocompatibility, degradability, noncytotoxic byproduct formation, and soft tissue mechanical properties, which provide the correct vascularization. Many research teams have thus studied natural, synthetic, and biological scaffolds (Table 1).

**Table 1:** Classification of different scaffolds based on features, production techniques

Class of materials	Materials	Scaffold production	Advantages	Issues
Natural materials *Similar properties to ECM, features can be modified by cross‐linking, biocompatibility and good cell attachment and proliferation*	Collagen	Injectable, microbeads, sponges, hydrogels	ASC differentiation and vascularization; possible addition of growth factors, modifiable porosity	Fast degradation, low mechanical properties
	Hyaluronic acid derivatives	Hydrogels, sponges	Good differentiation of adipocytes	Expensive
	Silk	Hydrogels, disks, thin films, sponges, tubes	Good mechanical properties, low immunogenicity, possible expression of growth factors and angiogenic factors	Unknown characteristics of degradation products
	Gelatin	Hydrogels, bioprinting, sponges	Good incorporation in natural tissue, possible addition of growth factors, usable together with other scaffolds	Fast degradation, low mechanical properties
Synthetic materials *Chemical and mechanical properties can be modified and have a good reproducibility*	PLGA	3‐D printing, hydrogels, sponges, injectable spheres	Biodegradable	Inflammation due to degradation products; short degradation time
	PCL	3‐D printing, electrospun meshes, sponges	Suitable mechanical properties; angiogenesis in ASC‐seeded and ASC‐unseeded constructs	Degradation time not well controlled; limitation in mammary cell attachment because of hydrophobicity
	Polyurethane	Sponges	Elastic and possible good angiogenesis and adipogenesis	Issues with in vivo transplant
	Polypropylene	Meshes	Biocompatibility	Not well absorbed
	Polylactic acid	Sponges	Good mechanical properties	Degradation time too fast
	PEG	Hydrogels	Water degradable, promotion of adipose tissue regeneration	Low mechanical properties, need for cross‐linking
Biological materials *Biological properties of ECM kept intact and decellularized adipose tissue leads to adipogenic differentiation without supplementation of differentiation factors*	Adipose‐decellularized ECM	Bioprinting, hydrogels, injectable microparticles, 3‐D printing	ECM provides right microenvironment for cells; well‐maintained 3‐D architecture also after decellularization	Not mass‐producible; technique is difficult and time‐consuming

*Note.* The same production techniques can be used for more than one type of material. The pros and cons of each scaffold material are summarized (O'Halloran et al., 2017; O'Halloran et al., 2018).

Abbreviations: ASC, adipose stem cells; ECM, extracellular matrix; PCL, polycaprolactone; PEG, poly (ethylene)glycol; PLGA, polylactic‐co‐glycolic acid.

Collagen, alginate, silk, gelatin, and hyaluronic acid derivatives are suitable natural materials for use as scaffolds for mammary gland tissue regeneration (O'Halloran et al., 2017; Zhu & Nelson, 2013). Collagen gels have been used as a 3‐D matrix on which preadipocytes were placed in a coculture with human autologous mammary epithelial cells (Wang et al., 2010). For example, researchers seeded a coculture of nonmalignant human epithelial cell lines (MCF10A) and predifferentiated hASCs, which were embedded in Matrigel and a 3‐D porous collagen scaffold (O'Halloran et al., 2017; Wang et al., 2009; Wang et al., 2010). They verified that the presence of hASCs increased both alveolar and ductal morphogenesis, also inhibiting epithelial cell proliferation. This was done in order to propose a regeneration therapy for restoring all the various types of tissue that make up the breast. In contrast to cocultures, Wang et al. (2009) also showed that monocultures can only regenerate the alveolar structures, which led to the analysis of cocultures.

Yao, Zhang, Lin, and Luan (2013) verified that collagen/alginate microspheres were suitable for ADSC encapsulation and delivery. After 4 weeks of using an adipogenic differentiation medium, from a macroscopic point of view, the microspheres were similar to the adipose tissue lobules. 3‐D silk scaffolds with hASCs and human umbilical vein endothelial cell cocultures depict human vascularized adipose tissue (Kang, Gimble, & Kaplan, 2009). Silk has a controllable degradation time, thanks to which it is possible to enhance neovascularization and prevent premature loss of nutrient transport through the matrices, which may lead to necrotic areas. This latter issue must be taken into account in choosing the best scaffold to degrade with a suitable kinetics mechanism.

A 2010 study used gelatin sponge scaffolds onto which human mammary stem cells were seeded. Thanks to the adipogenic differentiation medium, the authors observed the accumulation and expansion of lipid droplets (Wang et al., 2010).

Polylactic acid, polyglycolic acid, polyethylene terephthalate, polylactic‐co‐glycolic acid, polyurethane, and polycaprolactone are suitable synthetic materials for mammary gland tissue regeneration (Zhu & Nelson, 2013). These kinds of scaffolds, with adipogenic stimulants, have shown significant adipogenic transcript levels and substantial lipid accumulation, which unfortunately decreases in vivo (Zhu & Nelson, 2013).

Adipose decellularized extracellular matrix is the key ingredient for biological scaffolds. This type of scaffold has suitable biological properties and an excellent maintenance of its 3‐D architecture and biochemical composition, after decellularization.

Mature adipocytes were analyzed for 8 weeks after seeding within a decellularized human placenta scaffold. The results were positive; however, this procedure is expensive and time‐consuming (Flynn, Prestwich, Semple, & Woodhouse, 2009).

The production of the three categories of scaffolds described above is based on different techniques: 3‐D printing, sponge, hydrogel, and microbeads (O'Halloran et al., 2017). O'Halloran, Dolan, Kerin, Lowery, and Duffy (2018) concentrated on the characterization of biological, synthetic, and natural hydrogels because of their potential in regenerative therapies due to their particular characteristics. Hydrogels are 3‐D cross‐linked hydrophilic networks that absorb and maintain large quantities of fluids and incorporate live cells. In porous hydrogels, the pore dimension and density of porosity influence vascularization (Eghbali et al., 2017), which is needed for a real regenerative implantable adipose tissue; within nonporous hydrogels, degradation rate determines the correct vascularization. A study reported by O'Halloran and colleagues showed poly (ethylene)glycol diacrylate to create a hydrogel, which encapsulated bone marrow mesenchymal stem cells differentiated in an adipogenic medium. in vitro results were positive and demonstrated the presence of mature adipocytes after 4 weeks.

Another possibility, from a tissue model perspective, consists in using several types of scaffolds joined together in order to both maximize their advantages and minimize their criticalities.

Mauney et al. (2007) studied the potential of collagen and polylactic acid matrices, which can support adipogenesis in culture and also showed promising behavior in vivo. Other studies have also verified adipose tissue regeneration by both synthetic and natural scaffolds. Synthetic scaffolds guarantee the precise control of the material properties, whereas natural scaffolds have better biocompatibility and degradation features (Zhu & Nelson, 2013).

**Figure 4:**
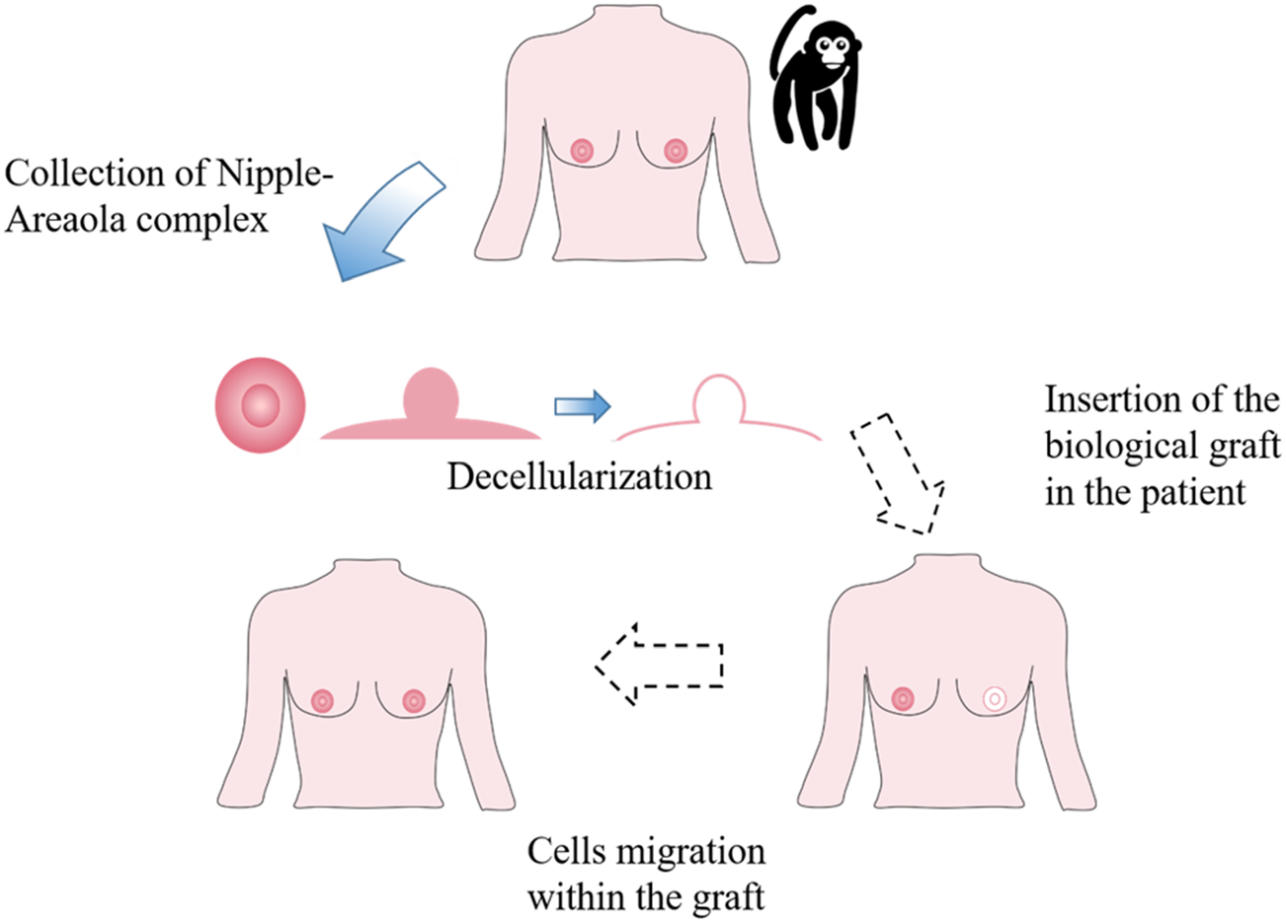
Nipple‐Areola Complex (NAC) Reconstruction. First the NAC is collected (either from a donor or the patient, while for the *in vitro* model, the rhesus macaque NAC was used). The NAC is then decellularized. The next steps (dashed arrows) are the NAC insertion in the patient and the cell migration within the graft

Not only has adipose tissue regeneration been studied on biological scaffolds. Pashos et al. (2017) described a tissue‐engineering approach for nipple‐areola complex reconstruction. They decellularized a whole donor NAC, maintaining the extracellular matrix structure unaltered (Figure 4). They found that the scaffold maintained good properties, which had a nonimmunogenic structure and the same micro architecture as a native NAC. The decellularized NAC also maintained the cell adhesion molecules (fibronectin and laminin). They employed their own protocol to conduct the study decellularizing the NAC tissues of rhesus macaque (Pashos et al., 2017).

They then conducted several analyses: genomic DNA, fragment and histological analysis and protein, GAGs, collagen, and elastin quantification. These produced good results in terms of efficient cell removal, DNA content reduction, and preservation of collagen fiber patterns. Lastly, they seeded the scaffold with bone marrow‐derived mesenchymal stem cells. They then have out proliferation and apoptosis analyses, demonstrating the bioactivity of the scaffold with cells migrating into the deeper portion in 7 days. In this time period, the cells were able to survive and proliferate; however, further in vivo studies are needed to prove the neovascularization process itself.

Finally, considering human iPSCs, Qu et al. (2017) employed a floating mixed Matrigel/Collagen I gel to embed human iPSC embryoid‐derived bodies. Collagen I was included in the 3‐D culture, due to the stiffness resembling that present in the normal mammary gland. The resulting alveolar structures observed in this 3‐D culture expressed breast, luminal, and basal markers, suggesting that mammary‐like cells are induced to differentiate. The results suggested mammary‐like differentiation from *human* iPSCs.

Despite the numerous advantages listed above, the principal limitation regarding adipose tissue regeneration in breast reconstruction is the difficulty of generating sufficient tissue volume to recreate the breast mound postmastectomy (O'Halloran et al., 2018).

### 2.3 Whole organ models

Due to the multiple cell types of the mammary gland, the major challenge is coculturing the epithelial cells, fibroblasts, endothelial cells, and adipocytes into a suitable matrix scaffold. Microfabrication approaches could lead then to the regeneration of a more complete mammary gland structure (Zhu & Nelson, 2013).

Organ‐on‐a‐chip platforms have been described in the literature, but for the sole purpose of studying the mammary gland and related breast cancer development (Campbell et al., 2014; Campbell, Hume, et al., 2014).

For example, a combination of adipose tissue engineering and microfabrication techniques has been proposed to build up a 3‐D engineered epithelium aimed at mimicking the physiological duct structure of the breast. The researchers started seeding preadipocyte in a collagen gel, in which they generated cavities using a stamp. They then induced the preadipocytes differentiation. Finally, epithelial cells covering the cavities formed tubules. They used this model to study gene expression, the mechanical stress profile during branching morphogenesis, and the consequences of a host microenvironment (Zhu & Nelson, 2013).

Future perspectives could focus on the potential of organ‐on‐a‐chip or a staminal niche (Raimondi et al., 2013) (to expand stem cells in vitro); however, researchers are still far from regenerating the whole organ architecture in vitro.

## 3 PRECLINICAL TRIALS

Animal models can represent human tissue and physiology; however, the efficacy and safety of newly developed strategies need to be demonstrated before clinical trials can occur. Preclinical inspections are thus mandatory (required by European law) on all human‐pledged drugs or cellular therapy products.

This section describes the potential of ADSCs to provide new adipogenesis and vasculogenesis, the oncological risks that entail examining the interaction between breast cancer cells, and fat grafting enriched with ADSCs.

### 3.1 ADSC effects on fat grafts

Autologous fat grafting is a well‐known technique for breast reconstruction; however, recent studies have reported reabsorption rates from 40% to 60% of the injected volume. Much of the volume loss is probably due to inadequate vascularization and to intolerance of the fat tissue to hypoxia (Tsuji et al., 2018). In addition, ADSCs are suspected of supporting the growth and the progression of tumors, which could increase the risk of breast cancer recurrence (Schweizer et al., 2015).

To investigate the effects of ADSCs on fat grafts, Jiang, Li, Duan, Dong, and Wang (2015) carried out animal experiments in which different techniques were used on 18 mice in the following groups: control, ADSCs, and ADSCs + basic fibroblast growth factor (bFGF) method. Mice in the control group were injected with 2 ml of aspirated fat and 0.1‐ml medium directly. Mice in the second group were coinjected with a mixture containing 1 × 10^6^ ADSCs with 2 ml of aspirated fat treated in 0.1‐ml medium. Mice in the third group were injected with a blend of 10^6^ ADSCs with 2 ml of isolated aspirated fat and 100‐U bFGF. The aspirated fat and ADSCs were isolated by liposuction from six healthy female human donors. The fat was injected subcutaneously into the mouse's back and, after 12 weeks, each fat graft was weighed, and the volume was measured in order to calculate the survival ratio of transplanted fat using the following formula: *survived volume*/*previous volume* (2 *ml*).

**Figure 5:**
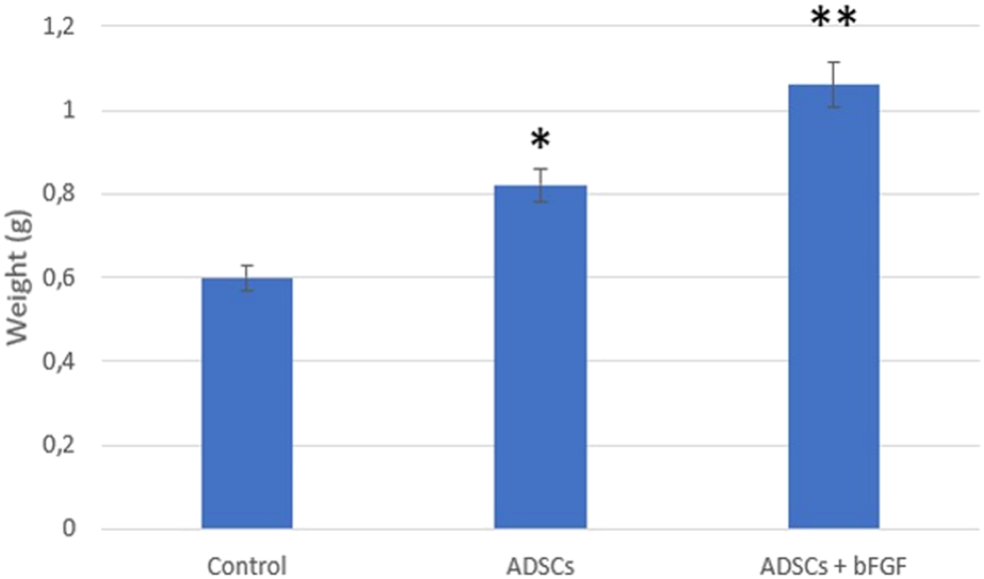
Weight in different fat grafts. *p < .01 versus the control group; **p < .05 versus ADSCs group, (Jiang et al., 2015). ADSCs, adipose‐derived stem cells; bFGF, basic fibroblast growth factor

**Figure 6:**
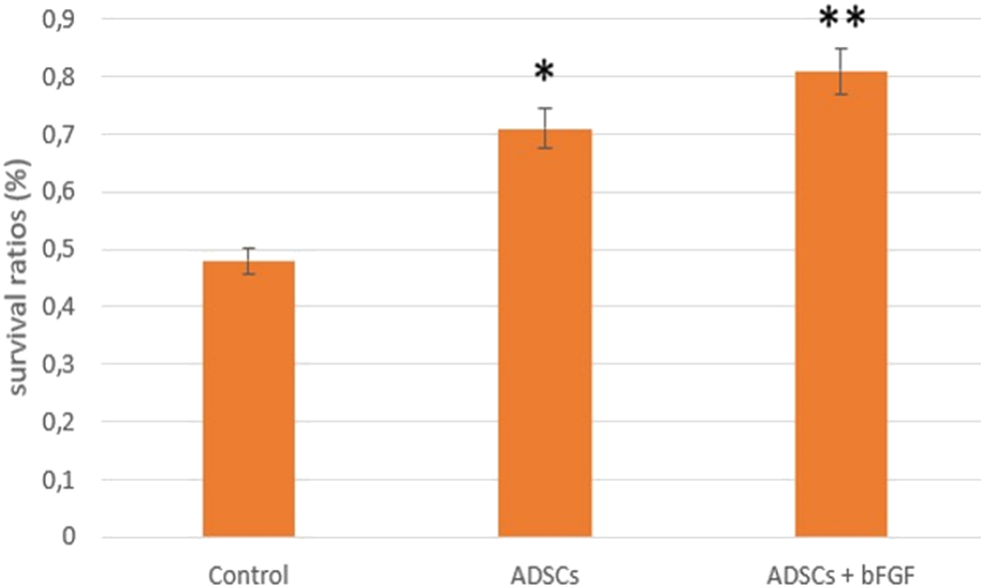
Survival ratio in different fat grafts. *p < .01 versus the control group; **p < .05 versus ADSCs group, (Jiang et al., 2015). ADSCs, adipose‐derived stem cells; bFGF, basic fibroblast growth factor

The results demonstrated that the weight and survival ratio in ADSCs and ADSCs + bFGF‐treated fat grafts were significantly higher than the control group (Figures 5 and 6).

Jiang's study demonstrated that ADSC treatment improves fat graft survival and fat integration. In particular, ADSCs + bFGF treatment had the best beneficial effect on the fat graft weight and survival ratio due to the ability of bFGF to modulate stem cell self‐renewal, differentiation, and survival.

### 3.2 ADSC effects on breast cancer cells

To study the risk of tumor formation and cancer metastasis, (Sun et al., 2009) transplanted breast cancer cell lines (MDA‐MB‐231) with and without ADSCs into two different mouse groups. The researchers isolated ADSCs from fresh human mammary fat tissue: The samples were harvested from women who underwent surgery for cosmetic reasons.

First, they injected 2 × 10^6^ MDA‐MB‐231 cells into the mammary fat pad of nonobese, diabetic immunodeficient mice, and after 22 days, they computed the tumor volume as $V=\frac{\text{length}\cdot {\text{width}}^{2}}{2}$. On Day 22, the authors performed an intravenous injection of 10^6^ human ADSCs in one of the groups. After 2 weeks, they calculated the dimension of the tumor in both groups, and the comparison of the results illustrated that the volume of cancer cells coadministered with ADSCs was significantly lower than the one with the MDA‐MBI‐231 cells alone. ADSCs were thus capable of inhibiting the growth of MDA‐MB‐231 by inducing their apoptosis. Because ADSCs may also promote angiogenesis, which can potentiate breast cancer cell metastasis, the authors measured the numbers of lung metastases in both groups. The number of lung metastases was reduced by 50% in the group in which ADSCs were injected. The researchers also analyzed the effects of ADSCs in the early stage of breast cancer, and the results showed that human ADSCs were able to inhibit tumor formation and progression in the group where these cells had been injected. However, these cell interactions did not show a comparable effect when ADSCs were administered intravenously in the mice (Sun et al., 2009).

To examine the interaction between breast cancer cells and the fat grafting enriched with ADSCs, (Rowan et al., 2014) performed xenograft procedures in which 15 mice were divided in three groups (*n* = 5 mice/group) by injecting either 3 × 10^6^ MDA‐MB‐231 human breast cancer cells, 3 × 10^6^ ADSCs, or MDA‐MB‐231 human breast cancer cells + ADSCs in 150 μl of phosphate‐buffered saline Matrigel mixture (50‐μl cell suspension in phosphate‐buffered saline was mixed with 100 μl of Matrigel) orthotopically and bilaterally into the inguinal mammary fat pads of mice.

ADSCs were isolated from the abdominal lipoaspirates from three female human donors with three different body mass indexes (BMI). However, MDA‐MB‐231 human breast cancer cells represent a model of “triple negative” breast cancer in patients, a more aggressive and metastatic breast cancer subtype. In all experiments, tumor measurements were taken twice a week.

**Figure 7:**
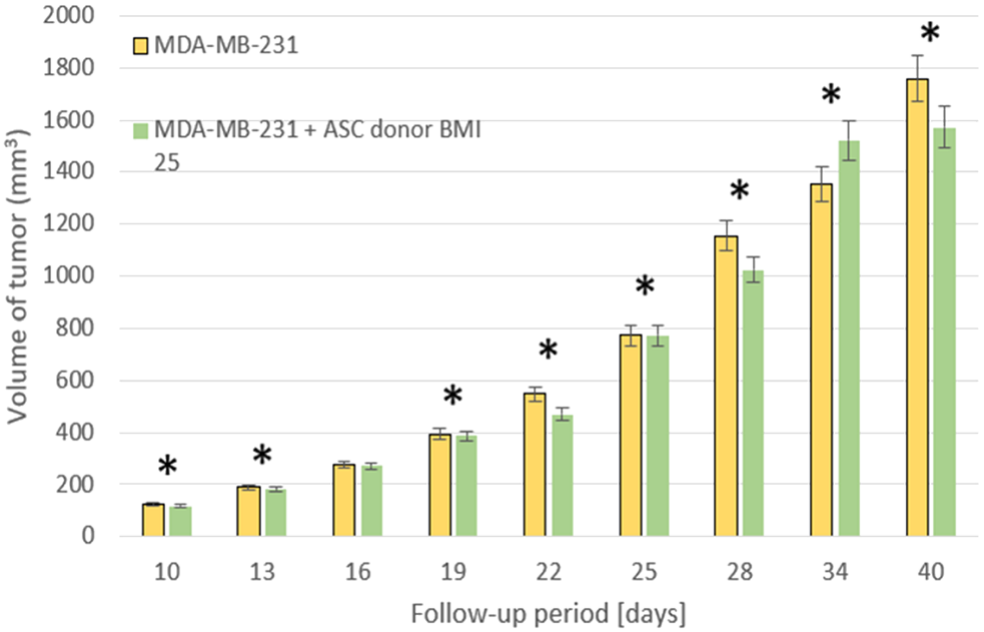
3 × 10^6^ human MDA‐MB‐231 breast cancer cell lines were injected subcutaneously into the mammary fat pads with or without 3 × 10^6^ human ASC cells from a donor with a body mass index of 25. *p < .05, (Rowan et al., 2014). ASC, adipose stem cells; BMI, body mass index

**Figure 8:**
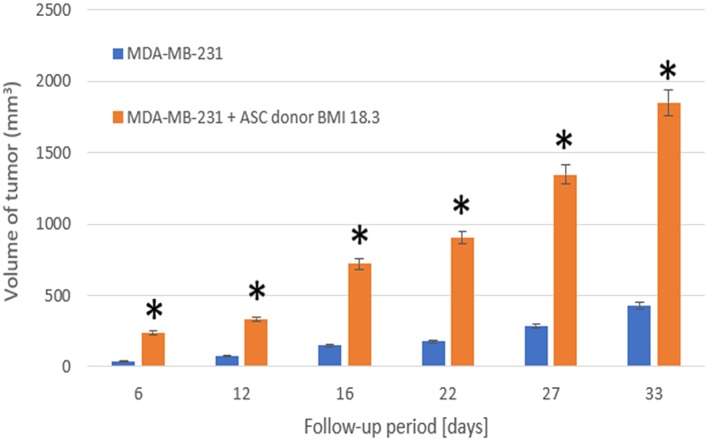
3 × 10^6^ human MDA‐MB‐231 breast cancer cell lines were injected subcutaneously into the mammary fat pads with or without 3 × 10^6^ human ASC cells from a donor with a body mass of 18.3. *p < .05, (Rowan et al., 2014). ASC, adipose stem cells; BMI, body mass index

The results showed that coinjection of BMI 25 ADSCs with MDA‐MB‐231 cells did not alter the primary tumor volume up to 40 days postinjection with tumor sizes that were comparable with MDA‐MB‐231 alone tumors (Figure 7). However, coinjection of ADSCs from the BMI 18.3 donor with MDA‐MB‐231 stimulated the growth of the tumors (Figure 8).

The presence of a significant donor‐dependent effect on the ability of ADSCs to stimulate primary MDA‐MB‐231 tumor growth was demonstrated; however, why this may be a crucial parameter for possible differentiation of the tumor cells is still unknown.

Tsuji and collaborators (Tsuji et al., 2018) applied two different approaches to model the interaction between tumor cells and fat grafting. In both approaches, the authors used MDA‐MB‐231 cancer cells and subcutaneously injected the fat grafts into the mammary fat pads of immune deficient mice and harvested the specimen 6 weeks postimplantation. In the first approach, the tumor cells were seeded into human fat grafts; in addition, Matrigel made up of only cancer cells was injected as a positive control. In the second approach, a simulation of breast reconstruction was carried out by injecting, into the same site, 100 μl of human adipose tissue, 2 weeks after the cancer cells had engrafted. Using regression analysis on the excised samples, results illustrated the survival of tumor cells in the fat graft. However, autologous fat grafts are not a favorable environment for the growth of breast cancer cells; in fact, ADSCs with MDA‐MB‐231 decreased the proliferation of the tumor and increased fibrosis. Thus, ADSCs may even have a repressive effect on tumor‐cell proliferation.

Table 2 summarizes the experimental studies conducted on ADSCs when these cells are coinjected in fat grafts together with the interactions between breast cancer cells and ADSCs. These studies revealed that ADSCs have the capacity to improve the long‐term retention of the grafts by reducing the reabsorption rate. However, the interaction between the breast cancer cells and the fat grafting enriched with ADSCs showed contradictory results, displaying both cancer‐suppressing and also cancer‐promoting effects.

**Table 2:** In Vivo experiments studying the potential of ADSCs when coinjected in fat grafts and the effects of ADSCs on breast cancer

Reference	Study model	Regeneration Strategy	Cells	Benefits	Risks	Therapy for
(Jiang et al., 2015)	Mice	Fat graft enriched with ADSCs and enriched with ADSCs+bFGF	ADSCs from human lipoaspirates and bFGF	Weight of the graft ↑ Survival ratio for graft↑	None	Breast augmentation
(Tsuji et al., 2018)	Mice	Fat graft enriched with ADSCs	ADSCs from human lipoaspirates MDA‐MB‐231, BT‐474 breast cancer cells	Tumor growth↓ Angiogenesis ↑	None	Breast augmentation after mastectomy
(Rowan et al., 2014)	Mice	Fat graft enriched with ADSCs	ADSCs from human lipoaspirates MDA‐MB‐231 breast cancer cells	Tumor growth ↔ if BMI = 25.0	Tumor growth ↔ if BMI=18.3	Breast augmentation after mastectomy
(Orecchioni et al., 2013)	Mice	Fat graft enriched with ADSCs	ADSCs from human lipoaspirates HCC1937, MDA‐MB‐436, ZR75‐1 breast cancer cells	None	Tumor growth ↑Metastatic spread ↑EMT ↑	Breast augmentation after mastectomy
(Martin‐Padura et al., 2012)	Mice	Fat graft enriched with ADSCs	Murine whole fat HMT‐3522 S3 (preinvasive), HMT‐3522 T4‐2 (invasive) MDA‐MB‐231 breast cancer cells	Angiogenesis ↑	Tumor growth ↑Metastatic Spread ↑	Breast augmentation after mastectomy
(Zimmerlin et al., 2011)	Mice	Fat graft enriched with ADSCs	Human abdominal whole fat Human MPE breast cancer cells	None	Tumor growth↑ (active cells, but not resting cells)	Breast augmentation after mastectomy
(Sun et al., 2009)	Mice	Fat graft enriched with ADSCs	Human breast whole fat MDA‐MB‐231 breast cancer cells	Tumor growth↓ Metastatic spread↓ No early carcinogenesis improvement	None	Breast augmentation after mastectomy

Abbreviations: ADSCs, adipose‐derived stem cells; BMI, body mass index; bFGF, basic fibroblast growth factor.

The investigation into the possible growth of the tumor due to the combination with ADSCs is still a great debate. Many factors and variables are possibly involved. According to Sun and colleagues, ADSCs seemed to have no effect when administered intravenously after the transplantation of cancer cells (Sun et al., 2009). Other factors could be the weight of the patients; thus, future studies using multiple donors with different BMIs will be able to determine whether donor BMI has an impact on the effects of ASCs on tumor growth (Rowan et al., 2014).

Although clinical trials have been carried out on the use of fat grafting combined with ADSCs, the data on the influence of ADSCs on breast cancer cells are still contrasting (Schweizer et al., 2015).

## 4 CLINICAL TRIALS

This section focuses on the results of six clinical trials (Table 3) on patients who underwent regenerative therapies essentially based on cell‐assisted lipotransfer procedures, instead of conventional treatments, such as mastectomy, breast conserving therapy, and pure autologous fat grafting.

**Table 3:** An outline of the main significant clinical trials found in the literature

Reference	Conventional Therapy and Indications (I)	Regenerative Therapy	No. of patients (no) Age (y, years)	Follow‐up (months)	Results (Phase 1)	Results (Phase II)	Complications (operative and in the follow‐up period)	Evaluation methods of the results
(Domenis et al., 2015)	‐Autologous fat grafting (A), breast reconstruction, breast tissue contour correctionI: Breast augmentation	e‐SVF (Enriched Stromal Vascular Fraction) fat grafting (B)	no.36:16 (A, control group)20 (B, experimental group) y. 21–71 (A)19–74 (B)	12	Long‐term augmentation effectDose: ≃10^6^ cells per milliliter of harvested fat tissue	Thicker fat layer around the mammary gland	None	Ultrasonography imaging
(Dos Anjos, Matas‐Palau, Mercader, Katz, & Llull, 2015)	‐Autologous fat grafting ‐Breast reconstruction, breast volume restoration I: Breast augmentation, breast cancer stage I to III	e‐SVF fat grafting	no.77 fat grafting:21 (control group, low dose of SVF), 56 (experimental group,high dose of SVF) y. 18–61	18	75% and 50% breast volume retention in high and low e‐SVF groups, respectively Dose: >2 × 10^5^ cells per mL of harvested fat tissue (high e‐SVF group); <5 × 10^4^ cells per milliliter of harvested fat tissue (low e‐SVF group)	Decrease in the early postsurgical breast edema; Improvement of long‐term volume retention	Mondor's diseasenine cases of subcutaneous benign lumps. 14 oil cysts; No intraoperative complications	Imaging 3‐D, 3‐D scanner, superimposed to measure difference.
(Gentile et al., 2012)	‐Autologous fat grafting (A) ‐Breast reconstruction, correction of contour and volume defects I: Breast cancer stage I to III	e‐SVF fat grafting (B), fat grafting with PRP (Platelet‐rich plasma) (C)	no.10 (A,C control group)n.13 (B,C experimentalgroups) y. 19–65	30	No microcalcifications No local tumor recurrence Dose: 200 mL average fat grafting for each breast. 5 × 10^4^ SVF per milliliter of harvested fat tissue (B); 0,.5 ml of PRP per milliliter of harvested fat tissue	More natural breast contour and softness; Higher augmentation effect; Minor volume loss	None	Team evaluation; Patient self‐evaluation
(Kamakura & Ito, 2011)	‐Autologous fat grafting ‐Breast augmentation I: Breast augmentation	Autologous adipose‐derived regenerative cells	no.20 Single‐Arm study. y. 21–52	12	Enhanced augmentation effect Dose: 240 ml average fat grafting for each breast; 3.42 × 10^5^ cells per gram of harvested fat tissue	Increase in the circumferential breast measurement (BRM)	Cyst formation in two patients	Volumetric measurements
(Pérez‐Cano et al., 2012)	‐Breast conserving therapy (BCT) ‐Breast tissue contour correction I: Breast augmentation, breast cancer stage I to III	ADSC	no.12 Single‐Arm study. y. 37–68	12	Permanent augmentation effectNo local tumor recurrenceNo post‐operative complicationsDose: 140 ml average fat grafting for each breast; 2.95 × 10^5^ ADRCs per milliliter of harvested fat tissue	Improvement in breast contour defects; More natural breast shape; Reduction in scar tethering	None	MRI imaging; Ultrasonography imaging; Likert Scale for the evaluation of breast defect and contour
(Yoshimura et al., 2010)	‐Mastectomy ‐Breast reconstruction or augmentation I: Breast augmentation	e‐SVF fat grafting	no.15 Single‐Arm study. y. 35–50	12	No microcalcifications Minimal volume lossPreservation of the fat tissueDose: 264 ml average fat grafting for each breast; 9.7 ± 1.7 × 10^7^ stromal vascular cells per liter of fat tissue	More natural breast softness; Symmetry; Thicker fat layer around the mammary gland	Slight post‐operative atrophy; Few cysts (<5 mm)	MRI imaging; Mammography; Photography; Videography; 3‐D imaging

*Note* **.** The letter “I” in the first column indicates the case in which the proposed approaches are clinically eligible.

Abbreviations: ADRC, adipose‐derived regenerative cell; ADSCs, adipose‐derived stem cells; SVF, stromal vascular fraction.

**Figure 9:**
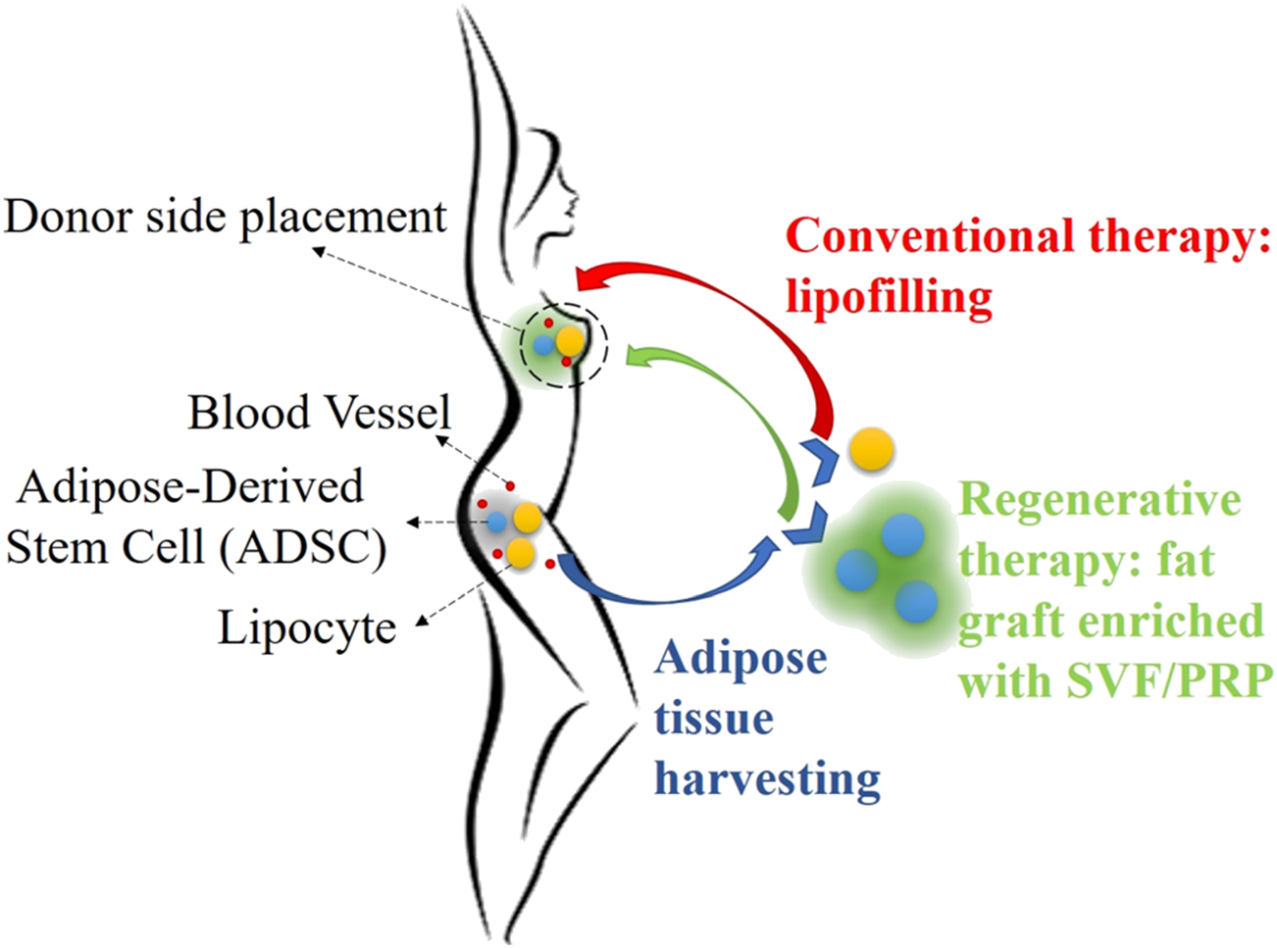
Cell assisted lipotransfer procedure: harvested fat is separated from the donor (the most common sites are the lower abdomen and inner thigh) and processed in one part (lipoaspirate) that is used as lipofilling and in another that is digested by enzymes in order to isolate stem cells (stromal vascular fraction, SVF) and expand them. Fat is then enriched with SVF or platelet‐rich plasma (PRP)

In the cell‐assisted lipotransfer method (Figure 9), which is performed in all the trials mentioned below, fat is enriched with ADSCs, contained in the SVF or with platelet‐rich plasma, obtained after enzymatic digestion of fat or after cell culture, to improve the fat survival rate. In all the alternative treatments, fat grafting works only as a decellularized scaffold, on which cells are seeded, as described in the section related to in vitro and animal trials.

All these clinical trials suggest that ADSCs can survive without undergoing postsurgery hypoxia, which is thought to result in the necrosis of a conventional fat graft (Khouri et al., 2014). This provides the scientific rationale for the use of these procedures in breast surgery and consequently makes these techniques key in the repertoire of techniques available to surgeons (Yoshimura et al., 2008). All the reported trials have a common primary objective, which consists in breast reconstruction or augmentation and, as secondary objective, esthetic improvement. In each trial, patients signed an informed consent regarding “the aims, methods, sources of funding, any possible conflicts of interest, institutional affiliations of the researchers, the anticipated benefits, and potential risks” (i.e., tumor induction) of the study and the discomfort it may derive, according to the Declaration of Helsinki emanated by the World Medical Association (“World Medical Association Declaration of Helsinki”, 2013).

In the following section, three significant clinical trials are reported; all of which should be considered as an attempt to find a solution to the disadvantages of each of the conventional therapies mentioned above.

### 4.1 Fat graft enrichment with SVF cells

In the context of fat graft enrichment, Yoshimura et al. analyzed 15 patients in a clinical single‐arm trial that reached Phase II. After breast implant removal, a cell‐assisted lipotransfer was carried out, based on the injection, into one single breast, of 264 ml of autologous adipose tissue extracted from the abdomen or thighs enhanced with 9.7 ± 1.7 × 10^7^ stem cells (SVF) per liter of tissue, isolated, expanded, and seeded in the fat graft (Yoshimura et al., 2006).

One quantitative parameter used to verify the efficacy of this therapy is the surviving fat volume, after a 1‐year follow‐up (ε[mL]), calculated as Equation (1):

(1) $$\varepsilon =V_{\text{removed implant}}+V_{\text{postoperative breast}}-V_{\text{preoperative breast}}$$

**Figure 10:**
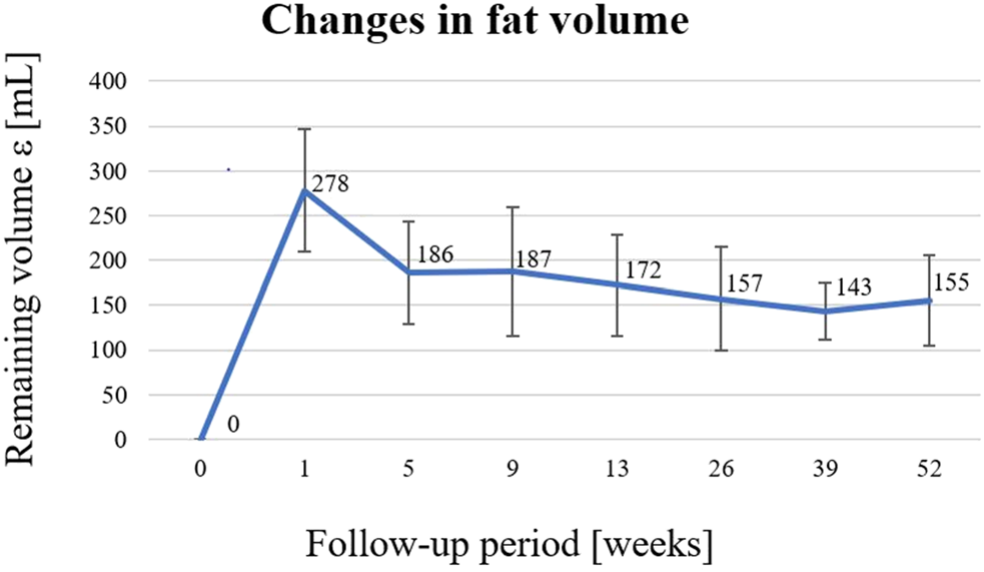
Average trend and standard deviation of the remaining fat volume for the right breast in a follow‐up period of 12 months. This parameter takes into account the volume of the removed prosthesis and the variations in breast volume (Yoshimura et al., 2006)

Yoshimura et al. used three‐dimensional measurement tools to quantify each volume of the expression above. The outcomes (Figure 10) show that the average surviving volume of the transplanted adipose tissue in the right breast varies during the follow‐up. In the first 5 weeks, there is a gradual absorption of fat, whereas in the remaining period, the volume decreases slightly, reaching 155 ± 50 ml, more than half of the injected adipose tissue.

No calcifications and microcalcifications were observed. In conclusion, these results suggest that the enrichment of fat grafting with SVF cells, in a cell‐assisted lipotransfer, may not be a safe and effective treatment, but might also be a suitable alternative to the breast reconstruction or augmentation (Yoshimura et al., 2010).

**Figure 11:**
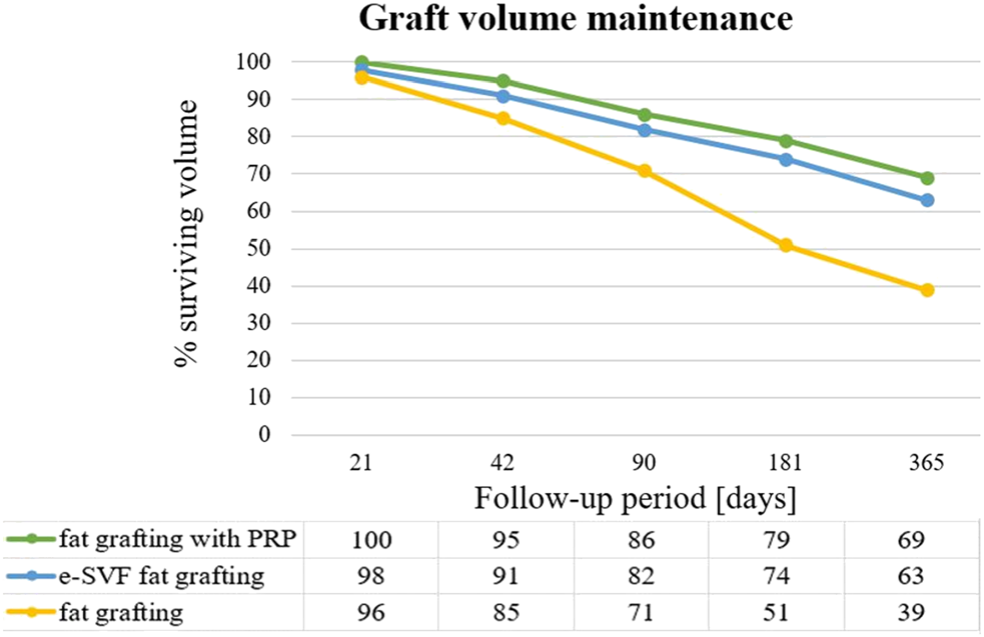
Trend of the remaining fat volume in a 1‐year follow‐up. The fat grafting with platelet‐rich plasma shows the best performance with respect to conventional fat grafting and enriched SVF fat grafting (Gentile et al., 2012). PRP, platelet‐rich plasma; SVF, stromal vascular fraction

**Figure 12:**
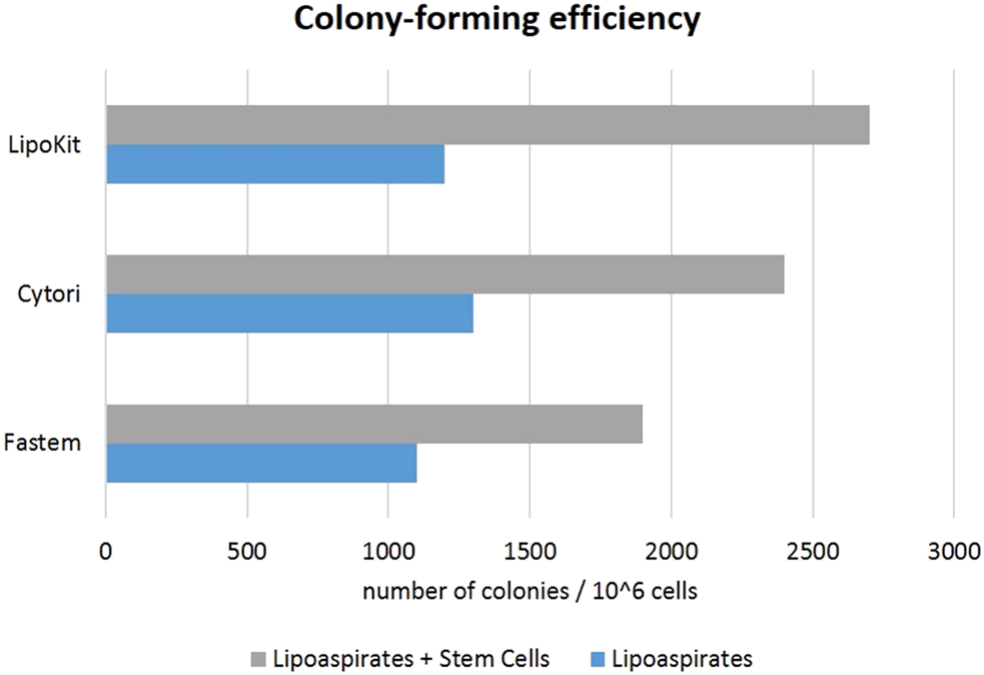
Number of colony‐forming units: comparison between adipose tissue alone and adipose tissue enriched with stem cells through three commercial devices (Domenis et al., 2015)

Considering grafts enhanced with SVF, another research group based in Italy (University of Rome “Tor Vergata”) carried out a clinical trial, first in Phase I and then in Phase II, in order to examine the defects of breast tissue, maintaining the natural volume of the mammary gland as much as possible (Gentile et al., 2012). Gentile et al. compared the effects of the conventional transplantation of only autologous centrifuged fat tissue (200 ml of harvested fat tissue: control group named A) with alternative treatments: SVF‐enhanced fat grafting (200 ml of harvested fat tissue enriched with 5 × 10^4^ stem cells per mL of tissue, group named B) and fat grafting with platelet‐rich plasma (200 ml of harvested fat tissue enriched with 0.5 ml of platelet‐rich plasma per milliliter tissue, group named C). The authors investigated the outcomes of the trial by evaluating the percentage of the remaining volume of transplanted tissue with respect to the injected one, after a 1‐year follow‐up. The results were analyzed by MRI imaging and are reported in Figure 12. Patients in groups B and C presented a relative percentage of 63% and 69% of the surviving fat volume, respectively. These values are successful compared with the 39% of remaining volume of patients who underwent the conventional therapy (Figure 11).

This clinical trial also shows only a minimal loss of adipose tissue and the absence of calcifications; only in one case was a small cyst and microcalcification found (Gentile et al., 2012).

### 4.2 Fat graft enrichment with autologous ADSCs

In terms of autologous ADSCs, Pérez‐Cano et al. conducted a prospective, multicenter single‐arm clinical trial that reached Phase II on 67 patients, who presented volume losses after breast conserving therapy. The main goal of this trial was essentially the same as the previous one, namely, the treatment of breast tissue defects in order to at least obtain a natural breast volume. The research group proposed the injection, in each breast, of 140 ml of autologous adipose tissue, enriched with 22.95 × 10^5^ of adipose‐derived regenerative cells per milliliter of harvested fat tissue with respect to a total lipoaspirate harvesting of 364 ml. At midfollow‐up (the 6th and 12th months), postoperative images reported that the breast tissue had remained stable in terms of the absence of exterior deformities. In addition, MRI images confirmed the improvement of the breast contour in 54 patients. Local cancer did not recur nor new diseases develop in any of the analyzed cases, which suggests the safety of the treatment (Pérez‐Cano et al., 2012).

All the clinical trials reported in Table 3 reached Phase II, except for the study conducted by a research group based in Italy. Through a randomized placebo‐controlled trial (Phase III), Domenis and collaborators, using lipotransfer techniques for breast reconstruction, concluded that autologous fat grafts enriched with cells could significantly contribute to the long‐term preservation of the transplanted adipose tissue. The cell‐assisted techniques used by Domenis, to apply the regenerative treatment, are also currently available on the market (Phase IV; Domenis et al., 2015).

In terms of safety and efficacy (Phase I), all these preliminary procedures have had good results; however, additional trials with longer follow‐up periods are essential for the evaluation of the possible risks introduced by these methods in several clinical circumstances.

## 5 COMMERCIAL PRODUCTS

With regards to regenerative therapies for breast reconstruction and/or augmentation, three devices are currently available on the market: Celution® 800/CRS, LipoKit II, and Fastem Coris (Table 4).

**Table 4:** Commercial products mainly for breast reconstruction and augmentation

Reference	Product name	Company	Country	Regeneration therapy	Main uses
(Domenis et al., 2015) http://www.cytori.com	Celution® 800/CRS	Cytori Therapeutics Inc.	Deeside, UK	Autologous fat grafting enriched with autologous ADSCs; Enzymatic isolation of ADSCs	Filler in breast augmentation; Aesthetic body contouring; Breast reconstruction; Preservation of breast function
(Domenis et al., 2015) http://www.medikanint.com	LipoKit II	Medikan International Inc.	Pusan, Korea	Autologous fat grafting enriched with autologous ADSCs; Enzymatic isolation of ADSCs	Filler in breast augmentation; Aesthetic body contouring; Breast reconstruction
[Domenis et al., 2015]	Fastem Corios	CORIOS Soc. Coop	San Giuliano Milanese, Italy	Autologous fat grafting enriched with autologous ADSCs; Mechanical isolation of ADSCs	Filler in breast augmentation; Esthetic body contouring; Breast reconstruction

Abbreviation: ADSCs, adipose‐derived stem cells.

The common procedure is based on the harvesting (liposuction) and centrifugation of autologous fat tissue, from which ADSCs are separated and extracted. The harvested cells are then expanded in specific culture conditions. Lastly, cells are seeded on the harvested adipose tissue, which is grafted on the patient's adipose tissue. Once in situ, cells start committing.

There are two main differences between the three devices: first, the method whereby stem cells get isolated from the tissue allowing them to aggregate in colony‐forming units; and second, the cell lineages toward which primitive cells commit. As regards the first method, Celution® 800/CRS and LipoKit II use an enzymatic separation of the SVF cells, whereas the Fastem Coris technique involves a mechanical procedure to carry out the same goal. Concerning the second method, Celution® 800/CRS allows cells to commit into adipocytes, epithelial cells, and muscle cells, LipoKit II enables differentiation into adipocytes, and the Fastem Coris technique does not allow significant commitment of cells, which tend to maintain a stable stemness phenotype.

Due to all these nonminimal manipulations (such as cells extraction and manipulation), these techniques are a feasible cellular therapy product alternative to conventional therapies, such as lipofilling (Figure 12).

A commonly employed parameter to quantify the efficacy of each system is the number of colony‐forming unit cells: an index of cell viability and an ability to differentiate (Domenis et al., 2015).

As reported in Figure 12, Celution® 800/CRS and LipoKit‐II‐enriched lipoaspirates are characterized by a higher number of colony‐forming unit cells, which is not so evident for the Fastem procedure.

## 6 CONCLUSIONS AND FUTURE PERSPECTIVES

The aim of regenerative medicine applied to breast tissue engineering is to overcome the main criticalities experienced with conventional therapies (mastectomy, breast conserving therapy, and lipofilling), regenerating fat tissue in the breast and improving the esthetic outcome (Visscher et al., 2017). To overcome the side effects of the standard treatments, a combination of lipofilling with tissue engineering has become a promising new technique.

Owing to the presence of multiple cell types in the breast tissue, mammary gland regeneration is quite complex. Although devices that exploit regenerative strategies are available on the market, the existing techniques are constantly being improved.

The key factor is the use of scaffolds, either biodegradable or integrated with the host tissue, to support cell growth until the formation of a stable and mature tissue.

In vitro models verify the importance of different cellular sources to regenerate both adipose and epithelial tissues. ADSCs have several advantages over other sources of stemness. They provide an accessible and easily available source of multipotent stem cells, and their harvest procedure is repeatable and safe, thanks to the negligible morbidity. Their capacity to differentiate both into adipocytes and epithelial cells makes them important for future applications in this field. Further studies are needed to verify the safety and efficacy of cells derived from ADSCs, before reaching preclinical and then clinical trials (Zhu & Nelson, 2013).

Finally, human iPSCs could be a promising cellular source. They are obtained by converting adult somatic cells of autologous origin, into pluripotent stem cells (Takahashi et al., 2007). This thus avoids ethical and technological controversies that hinder the application of human embryonic stem cells. A recent promising study demonstrated the possibility of obtaining mammary‐like cells and organoids from human iPSCs (Qu et al., 2017). Future research by this group will further examine the effects of growth factors on iPSC differentiation into mammary cells to refine the generation of a two‐layer mammary ductal structure. However, the research work is still in the in vitro phase; thus, many other studies are required before obtaining preclinical models. Moreover, criticalities related to the teratoma formation after reinfusion of differentiated cells from iPSCs need to be examined.

Many scaffolds have shown suitable properties as found in in vitro research. The main advantage of this strategy is that biomaterials already approved by the Food and Drug Administration, or other regulatory organisms (e.g., European Medicines Agency), can be adapted to create brand new cellularized constructs, which would speed up the development of all trials up to the commercialization phase (Jewell, Daunch, Bengtson, & Mortarino, 2015; Visscher et al., 2017).

It is worth noting that the principal purpose is not to replicate the physical structure, but rather promoting tissue regeneration by designing a customized scaffold architecture and using the patient's body as a bioreactor.

Adipose tissue has a high metabolic function; thus, the energy demand needs to be met, preventing any necrosis and volume loss caused by lipofilling procedures. The challenge is to develop new techniques involving customized scaffolds that can induce the vascular network, which can satisfy the adipose tissue metabolic demand, before the injection of the lipoaspirate (Visscher et al., 2017).

Among the scaffold‐based techniques, the fact that injectable hydrogels can be injected through minimally invasive techniques makes them an important option.

Various preclinical studies state that the use of an autologous fat graft enriched with ADSCs may improve the efficacy of fat transplantation. The results demonstrated that ADSCs promote vasculogenesis, angiogenesis, and blood vessel remodeling. Several angiogenic factors have the ability to stimulate microvascular endothelial cell proliferation thus promoting angiogenesis (Pérez‐Cano et al., 2012).

In addition, other studies have combined an autologous fat graft enriched with ADSCs and bFGF. The results highlighted that bFGF‐treated fat grafts improve the survival rate, vascularization, and angiogenic factor expression compared with grafts enriched with ADSCs only. The outcomes indicate that bFGF together with ADSCs can improve the efficacy of autologous fat transplantation and increase blood vessel generation involved in the benefits from bFGF (Jiang et al., 2015).

First, new regenerative techniques need to take into consideration the patient's safety, preventing regrowth of the tumor. Animal trials mostly focus on the interaction between the fat tissue and breast cancer cells because the relationship is still not well understood, and the results of these studies are sometimes not in agreement with clinical data. On the assumption of the worst‐case scenario of breast cancer cells left in situ, a fat graft injection post mastectomy has been modeled in rodents, which showed that the autologous fat graft is not a supportive environment for the growth of breast cancer cells. Although cancer cells survive and proliferate in fat grafts, the results indicate that fat grafting did not accelerate cancer cell proliferation and decreased the number of live tumor cells (Tsuji et al., 2018). Contradictory results on the other hand, have shown that human ADSCs harvested from patients with a body mass index of 18.3, and injected subcutaneously into the mammary fat pad of mice, promoted the growth and proliferation of breast cancer cells; thus, the weight of the patient can be a factor for cancer‐promoting effects. Further studies, using different BMIs, are needed to study the effect of ADSCs on tumor growth (Rowan et al., 2014).

Despite the occasional unsatisfactory results obtained in preclinical trials, some regenerative strategies reach the stage of human trials, most using the stromal vascular fraction to enrich autologous fat tissue (e‐SVF). The common target of these procedures is the long‐term assessment of the actual effects of SVF cells on fat graft volume retention and its maintenance.

Optimal results have been obtained, in particular for the enrichment of adipose tissue with platelet‐rich plasma cells, which increase the number of ASCs, maintaining the expression of CD34, an indicator of stemness (Gentile et al., 2012; Yoshimura et al., 2006).

Almost all patients involved in these trials were pleased with the esthetic outcome of the treatment, in terms of breast volume and contour preservation. The improved survival of breast tissue may be due to the ability of SVF cells to increase vascularization and secrete pro‐surviving growth factors. The crucial result is the absence of tumor recurrence in those patients who have undergone regenerative therapy due to breast cancer.

The overall results also highlight the superiority of regenerative therapies over standard procedures in obtaining both reliable and esthetically effective outcomes.

Clear evidence of the progress made by regenerative medicine in terms of breast tissue treatment is the availability on the market of three devices exploiting the cell‐assisted lipotransfer technique: Celution® 800/CRS, LipoKit II, and Fastem Coris.

In conclusion, breast tissue engineering can be considered as a viable alternative to the criticalities related to conventional therapies, potentially leading to a personalized medicine paradigm (Visscher et al., 2017).

## CONFLICT OF INTEREST

The authors have no conflicts of interest to disclose.
